# Nerve Growth Factor Receptor (NGFR/p75NTR) of the Small‐Spotted Catshark (*Scyliorhinus canicula*): Evolutionary Conservation and Brain Function

**DOI:** 10.1002/cne.70049

**Published:** 2025-04-12

**Authors:** Elena Chiavacci, Roberta Camera, Mario Costa, Baldassare Fronte, Eva Terzibasi Tozzini, Alessandro Cellerino

**Affiliations:** ^1^ Biology Laboratory (BIO@SNS) Scuola Normale Superiore Pisa Italy; ^2^ Neuroscience Institute National Research Council (CNR) Pisa Italy; ^3^ Department of Veterinary Sciences University of Pisa Pisa Italy; ^4^ Biology and Evolution of Marine Organisms Department (BEOM) Stazione Zoologica Anton Dohrn Napoli Italy; ^5^ Fritz Lipmann Institute for Age Research Leibniz Institute Jena Germany

**Keywords:** catshark, dogfish, nerve growth factor receptor, neurotrophins, nerve growth factor (NGF), p75, p75NTR

## Abstract

The p75NTR receptor, a member of the tumor necrosis factor (TNF) receptor superfamily, can participate in signaling pathways either by forming heteromeric complexes with other receptors, such as the Trk family (tropomyosin receptor kinases), or by functioning independently. p75NTR was investigated prevalently in the brain and retina of mammals, whereas almost nothing is known about its conservation among species. Here, we reconstructed the phylogenetic arb of p75NTR and described for the first time the p75NTR expression in the brain of the basal vertebrate Chondrichthyan *Scyliorhinus canicula* (*S. canicula*), uncovering the existing parallelism between ancient vertebrates and mammals. p75NTR functional conservation among vertebrates was further investigated by cloning the *S. canicula* nerve growth factor (NGF) and performing the canonical posterior commissure (PC)‐12 differentiation assay, which results in standard neurite‐like production. We then investigated the *S. canicula* p75NTR, which proves to be capable of complementing a specific clone of PC‐12 lacking p75NTR (PC‐12 p75NTR^−^/^−^). All together, our results highlighted the expression and functional conservation of p75NTR among vertebrates during the evolution.

## Graphical abstract

p75NTR expression in the brain of the basal vertebrate *Scyliorhinus canicula* revealed its conservation across species. Functional conservation among vertebrates was demonstrated (i) by performing the canonical PC‐12 differentiation assay with the *S. canicula* NGF and (ii) by complementing the p75NTR absence in a PC‐12 p75NTR^−^/^−^ knock‐out clone with the *S. canicula* p75NTR.

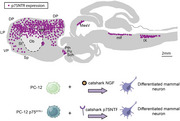

**Abbreviations**
BSA: basal superficial area
Gr: cerebellar granular layer
LP: lateral pallium
MesV: mesencephalic trigeminal nucleus
mlf: medial longitudinal fascicle
MP: medial pallium
OT: optic tectum
PC: posterior commissure
Po: preoptic area
Pt: pretectum
Pth: prethalamus
Sch: suprachiasmatic nucleus
SR: septal region
St: striatum
Th: thalamus
VM: mesencephalic motoneurons
VP: ventral pallium
Xm: vagal motor nucleus

## 1 Introduction

Nerve growth factor (NGF) is the prototype of the neurotrophin gene family and was originally defined for its ability to promote neurite formation from sensory neurons of the dorsal (DP) root ganglia. In 1979, two distinct NGF binding sites, one with high affinity and one with low affinity, were described on sensory neurons of chicken embryos (Sutter et al. 1979). Chao et al. (1986) and Radeke et al. (1987) clone a cDNA capable of producing a low‐affinity binding site that was termed p75 receptor (p75NTR). Later, the TrkA (tropomyosin‐related kinase receptor Type I) was identified as the higher molecular weight receptor for NGF (Kaplan et al. 1991; Klein et al. 1991). Although both TrkA and p75NTR can individually bind NGF to elicit independent signaling events, there is substantial evidence that the two proteins may cooperate, and a complex of both p75NTR and TrkA is necessary to obtain the high‐affinity binding site for NGF (Hempstead et al. 1991).

p75NTR is a member of the tumor necrosis factor gene family that can signal independently of Trk receptors and can form heteromeric complexes in combination with other interactors. In combination with sortilin, p75NTR binds proNGF and induces an apoptotic signaling pathway through its death domain (Nykjaer et al. 2004; Dasgupta et al. 2024). A number of other different p75NTR interactors have been identified, and through these interactions, p75NTR regulates a wide range of cellular functions, including programmed cell death, axonal growth, degeneration and regeneration, cell proliferation, myelination, and synaptic plasticity. The multiplicity of cellular functions governed by the receptor arises from the variety of ligands and co‐receptors that associate with p75NTR and regulate its signaling (reviewed in Malik et al. (2021)). For example, in association with LINGO‐1 and Nogo‐A, it participates in myelin‐dependent inhibition of axonal growth (Mi et al. 2004). On other hand, in interaction with Trks, it promotes survival, axonal growth, and differentiation (Reichardt 2006; Kraemer et al. 2014). Notably, small molecule inhibitors of neutrophil binding to p75NTR are emerging as possible targets for neurodegenerative diseases (Qian et al. 2019; Yang et al. 2020).

During development, the p75NTR is widely expressed in the nervous system where it regulates neuronal differentiation, migration, and axonal outgrowth. p75NTR also mediates the survival and death of newly born neurons, with functional outcomes being dependent on both timing and cellular context, recently Meier et al. (2019) demonstrate that p75NTR is required for the survival of neuronal progenitors and normal formation of the basal forebrain, striatum (St), thalamus (Th), and neocortex in mouse. In the adult nervous system, the expression of both p75NTR and TrkA is limited to specific neuronal populations such as the cholinergic neurons of the basal forebrain where they are co‐expressed. This is in contrast with the widespread expression of TrkB and TrkC, indicating that in the adult brain there is a preferential association of p75NTR with TrkA. NGF is known to play a central role in the trophism of telencephalic cholinergic neurons (Chen et al. 1997). Some populations of central neurons appear to express p75NTR in the absence of TrkA. One prominent example is the neurons of the mesencephalic complex of the trigeminal nerve, where binding of NGF to p75NTR induces neuronal death (Davey and Davies 1998). Another population where effects of p75NTR on neuronal survival in vivo are well‐characterized are the Müller glia cells of the retina. These cells express high levels of p75NTR, and binding of NGF abrogates the pro‐survival effects of NGF on damaged retinal ganglion cells (Lebrun‐Julien et al. 2009) and impairs survival of photoreceptors after light injury (Harada et al. 2000).

Up to date p75NTR was investigated prevalently in the brain and retina of mammals, with some information coming from other organism such as chicken (Sutter et al. 1979; Das et al. 1997), whereas almost nothing is known regarding its evolution and functional conservation among species. To address this gap in the current knowledge, we decided to focus our investigation on the small spotted catshark (*Scyliorhinus canicula*). The *S. canicula*, also known as the sandy dogfish, lesser‐spotted dogfish, rough‐hound, or morgay, offers several key advantages when it comes to study the evolution of p75NTR and its role in mediating the neurotrophic signaling. The most notable and valuable feature of *S. canicula* is that it belongs to the Chondrichthyes. Chondrichthyes are situated in a key strategic phylogenetic position of all other living jawed vertebrates (gnathostomes), being their outgroup. Importantly, the *S. canicula* is the first and most basal vertebrate reported to possess the full set of four neurotrophins, namely, the orthologs of the four mammalian neurotrophins BDNF, NGF, NT‐3, and NT‐4 (Chiavacci et al. 2023). Interestingly, the cephalic index of Chondrichthyes is larger than in teleosts, resulting in a cephalic index more similar to that of birds and mammals, whereas the allometric scaling among the different brain divisions is identical to that of primates (Northcutt 1977; Yopak et al. 2010). Other valuable advantages of *S. canicula* are its common presence in the European North Atlantic and the Mediterranean Sea, ease of collection, and possibility of captive breeding. All together, these enticing features are increasing the popularity of *S. canicula* as model system and qualifying it as the best model system for the study of p75NTR conservation in the brain.

## 2 Materials and Methods

### 2.1 Phylogenetic Analysis

Inference of p75NTR evolutionary relationships was performed using p75NTR peptidic sequences of different species obtained from NCBI https://www.ncbi.nlm.nih.gov/ and Ensembl genome browser 106 https://www.ensembl.org/index.html: Entries are listed in Table 1. The phylogenetic trees were constructed with MEGA11 (Molecular Evolutionary Genetics Analysis version 11) https://www.megasoftware.net/ as follows: Sequences of p75NTR were aligned with ClustalW, and the maximum likelihood methods were applied to the JTT + G model, whereas cladograms and robustness were estimated at each branching node by 100 random bootstrap replications. The phylogenetic view of p75NTR evolutionary relationships was obtained by MEGA11 as output.

**TABLE 1:** Species with respective entry IDs for the phylogenetic tree construction.

Species	Entry ID
*Mus musculus*	NP_150086.2
*Homo sapiens*	NP_002498.1
*Gallus gallus*	NP_001139605.2
*XP_005301977.2*	XP_005301977.2
*Zootoca vivipara*	XP_034991839.1
*Xenopus laevis*	NP_001081935.1
*Xenopus laevis*	XP_018093536.1
*Danio rerio*	NP_001185589.1
*Danio rerio*	XP_003198133.2
*Callorhinchus milii*	XP_042199211.1
*Amblyraja radiata*	XP_032891264.1
*Scyliorhinus canicula*	XP_038635035.1
*Chiloscyllium plagiosum*	XP_043531128.1
*Petromyzon marinus*	XP_032815093.1
*Branchiostoma floridae*	XP_035661065.1
*Strongylocentrotus purpuratus*	XP_030830229.1
*Eptatretus burgeri*	ENSEBUT00000003601.1

### 2.2 Tissues Processing

Adult specimens of *S. canicula* were supplied alive by local fishermen, and the animals were sacrificed, and brains were immediately dissected and fixed overnight in paraformaldehyde (PFA) 4% in compliance and approved by the Italian Ministry of Health (Cod. B290E.N.TU2). For the free‐floating in situ hybridization (ff‐ISH), following decapitation, the brains were dissected from the skull, post‐fixed overnight (ON) in PFA at 4°C in PBS, rinsed twice in PBS, and then equilibrated in sucrose 25% until the tissue sank to the tube bottom (a minimum of 6 h). The tissues were then embedded in Tissue‐Tek O.C.T. Compound (Sakura), and 100 µm thick sections were cut on microscope glass slides Menzel‐Glaser (Thermo Fisher Scientific). Slides with sections were stored at −20°C until hybridization. For RNA extraction, the fresh dissected brain was sunk immediately after dissection in QIAzol (Qiagen) and stored at −80°C until extraction.

### 2.3 Total RNA Extraction, cDNA, and RT‐qPCR

Total RNA was extracted using the RNeasy Mini kit (Qiagen) according to the manufacturer's protocol and quantified using a FC‐3100 (NanoReady) spectrophotometer; quality was checked by electrophoresis on agarose gel in RNAse‐free conditions. For cloning purposes, 1 µg of RNA was retrotranscribed with GoScript Reverse Transcription System (Promega), specific for long cDNA transcript production. For qRT‐PCR, 1 µg of RNA was retrotranscribed for cDNA synthesis with QuantiTect Reverse Transcription Kit (Qiagen), which provide an ad hoc DNAse treatment step to avoid genomic contaminations. qPCR was performed with SsoAdvanced Universal SYBR Green Supermix (Biorad) and 30 ng of cDNA for each sample as template in Rotorgene equipped with Rotorgene Q software (Qiagen). Relative gene quantification was calculated using the ΔΔCt method with *tbp* as reference gene. Primer sequences are *tbp* Fw: 5′‐AGACAATAGCCCTTCGAGCA‐3′, *tbp* Re: 5′‐TTCTTGCAGCCAATCGTGAC‐3′, *bdnf* Fw: 5′‐GTGAGCGTCCTGGAGAAGAT‐3′, *bdnf* Re: 5′‐TATCCATAGTTAGGGCGCGC‐3′, *p75NTR* Fw: 5′‐GGGATTTGTGCGGTTCCTTT‐3′, and *p75NTR* Re: 5′‐TACTGTCCAGGCATTGCTGA‐3′.

### 2.4 DIG‐Labeled Riboprobe Synthesis

The template was obtained by PCR amplification from cDNA using a forward Fw: 5′‐ATGGGATTTGTGCGGTTCCT‐3′ and a reverse primer carrying a T7 promoter sequence, here underlined, on its 5′ end, Re: 5′‐TAATACGACTCACTATAGGGCTGTTGTTGGAACTATTTTC‐3′. The PCR product was purified with Wizard SV Gel and PCR Clean‐Up System (Promega), verified via Sanger Sequencing (Eurofins Genomics), then 50 ng were used as template to be directly transcribed with T7 RNA polymerase (Thermo Fisher Scientific) and digoxygenated RNTP mix (Roche) for 2 h at 37°C. The resulting DIG‐labeled riboprobe was precipitated with 1/10 of volume of LiCl (5 M) and 2.5 volumes of ethanol ON at −20°C, washed with 75% ethanol, resuspended in nuclease‐free water, and stored at −80°C.

### 2.5 Free‐Floating In Situ Hybridization

ff‐ISH was performed according to Thisse and Thisse (2008) with some modifications as follows: Sections of 100 µm were rehydrated in PBS, detached from the glass slice and recovered in 2 mL Safelock Eppendorf, one section each. Sections were directly pre‐hybridized 30 min at 66°C and then incubated with digoxigenin DIG‐labeled probe at 66°C ON. Immediately before incubation, the probe was denatured at 80°C for 3 min. Sections were washed for 15 min at 66°C twice, first with SSC‐2x and then with SSC‐0.2x. Three washes in PBST at room temperature (RT) followed, then sections were incubated with anti‐Dig AP Fab (Roche) 1/2000 4°C ON in blocking solution (Roche). The day after, sections were washed 3 times for 5 min in PBST at RT and then moved in 24‐well, 1 section each well. Sections were treated with TMN solution (Tris‐MgCl2‐NaCl buffer) three times for 5 min, then stained with BM‐Purple (Roche). The staining was constantly monitored under stereomicroscope (M80 Leica) equipped with LED light O‐ring and blocked by washing in PBST. Once the color was fully developed, sections were postfixed in PFA 4% ON at 4°C and nuclei were stained with Hoechst 1/5000 5 min. Sections were mounted on Superfrost Plus glass slides (Thermo Fisher Scientific) with Fluoroshield Mounting Medium and coverslipped.

### 2.6 Immunohistochemistry (IHC)

All the immunohistochemical procedures were performed on ff‐ISH sections. After the developing of DIG signal, the sections were postfixed in 4% PFA ON at 4°C and then washed three times in PBS 1x. Sections can be stored several days in PBS at 4°C. For IHC, sections were permeabilized with tritonX 0.1% and blocked in basal superficial area (BSA) 5%, tritonX 0.1%. Primary antibodies were all incubated overnight at 4°C according to the following dilutions: anti‐tyrosine hydroxylase 1:500 (Abcam monoclonal rabbit [EP1533Y] Cat# ab75875, RRID:AB_1310786), anti‐NeuN 1:500 (Abcam monoclonal rabbit [EPR12763] Cat# ab177487, RRID:AB_2532109), anti‐S100‐beta 1:500 (Genetex polyclonal rabbit Cat# GTX129573, RRID:AB_2886037). For the double same host IHC, after the incubation with NeuN sections, they were washed three times in PBS 1x and incubated for 2 h at RT with Alexa Fluor 488‐AffiniPure Fab Fragment Rabbit Anti‐Goat IgG (H + L) (Jackson ImmunoResearch Labs Cat# 305‐547‐003, RRID:AB_2339544) diluted 1:400, according to manufacturer instruction. After three washes in PBS, sections were then incubated ON at 4°C with S100‐beta primary antibody as previously described. Secondary antibodies were incubated for 2 h at RT 1:500 (Invitrogen polyclonal rabbit Alexa Fluor 488 Conjugated Cat# A‐11008, RRID:AB_143165; Invitrogen polyclonal rabbit Alexa Fluor 568, Cat# A‐11011, RRID:AB_143157). Nuclei were stained with Hoechst 1/5000 5 min, and sections were mounted on Superfrost Plus glass slides (Thermo Fisher Scientific) with Fluoroshield Mounting Medium.

### 2.7 ff‐ISH and IHC Imaging

ff‐ISH whole panoramic view images were acquired with stereomicroscope (M80 Leica) equipped with Axiocam ERc 5S (Zeiss) and LED light O‐ring. ff‐ISH magnifications and IHC fluorescence images were acquired using epifluorescence microscope (Nikon, Eclipse 600) equipped with DS‐Fi3 color camera (Nikon) supplied with a double LED light O‐ring. Images were processed with Gimp 2.10.32 and ImageJ software.

### 2.8 Plasmids

pEC213 ScaNGF expression vector was built as follows: *S. canicula* NGF sequence was recovered from NCBI (Refseq XM_038820917.1) and gBlock synthetized (IDT) with the adding of KpnI and AgeI sites for the cloning in the pP2A‐mCherry‐N1 (Addgene‐plasmid #84329), harboring the multicloning site, the CMV promoter, and a T2A‐driven mCherry fluorophore. pEC207 p75NTR expression vector was built as follows: *S. canicula* p75NTR was cloned from *S. canicula* cDNA (see “Total RNA extraction, cDNA and RT‐qPCR” section) with the adding of KpnI and AgeI sites for the cloning in the pP2A‐mCherry‐N1 (Addgene‐plasmid #84329). As control, the PCS2‐EGFP vector, harboring the EGFP under the control of CMV promoter, was used.

### 2.9 Posterior Commissure (PC)‐12 Cell Differentiation and Complementation Assays

Rat pheochromocytoma PC‐12 cell lines were maintained at 37°C and 5% CO_2_ in DMEM medium (Invitrogen, Monza, Italy) supplemented with 10% horse serum (HS), 5% fetal bovine serum (FBS), 1% penicillin/streptomycin, 1% l‐glutamine (Gibco‐ThermoFisher, Monza, Italy), and grown as monolayer cultures according to ATCC standard protocols. Cells were plated at 80%–90% confluence in 6 cm diameter Petri dishes. For the dose‐dependent assay, PC‐12 wild‐type and PC12 p75^−^/^−^ cells were plated in parallel into 24‐well Primaria plates at low density of 15,000 cells per well. Differentiation was induced as described in Testa et al. (2022) with some modifications: Cells were starved in 1% HS + 0.5% FBS for 24 h, then medium was supplied with human NGF protein produced in‐house as previously described (Rattenholl et al. 2001; Paoletti et al. 2009) at the concentrations of 5, 10, 20, 50, and 100 ng/mL. Exposure to starving medium alone was used as control, and each condition was carried out in duplicates. Cells were imaged after 5 days post treatment using the Eclipse Ts2R inverted fluorescence microscope (Nikon) at 20× magnification, and nine different areas per well were acquired. Differentiation morphological analysis was performed as follows: One neurite‐like process was counted and the length of the longest neurite‐like process for each cell was measured using ImageJ software with the operator being blind to the cell genotype. Statistical analyses were performed using GraphPad Prism 8.0.2. A two‐way analysis of variance (ANOVA) was performed to evaluate the effects of PC12 cell clone and NGF concentration on neurite length. Post hoc comparisons were performed using the Bonferroni test to adjust for multiple comparisons. Results were considered statistically significant at *p* < 0.05.

For the complementation assay, differentiation assays were performed as described above with some modifications. Briefly, cells were starved in 1% HS + 0.5% FBS for 24 h, then reverse transfected with FuGENE HD (Promega) according to the manufacturer's instructions into 24‐well Primaria plates at low density. one day post‐transfection (dpt) cell were monitored for the reporter fluorophore presence by fluorescence microscopy with Eclipse Ts2R inverted fluorescence microscope (Nikon) to check the effectiveness of transfection. Subsequently, the transfection medium was replaced with the starving medium supplemented with human NGF protein produced in‐house as previously described (Rattenholl et al. 2001; Paoletti et al. 2009) at the concentration of 10 ng/mL. Cells were then live‐imaged after 5 dpt with Leica STELLARIS 5 inverted confocal microscope (Leica Microsystems CMS GmbH, Germany). Only fluorescent cells presenting at least one neurite‐like process were counted, and the length of the longest neurite‐like process for each cell was measured using ImageJ software. Experiments were performed in biological triplicates, and at least *n* > 104 cells were counted for each replicate. Statistical analyses were performed using GraphPad Prism 6.0, Student *t*‐test was performed between means for each treatment, and results were considered statistically significant at *p* < 0.05.

## 3 Results

### 3.1 Sequence of p75NTR in *S. canicula*

**FIGURE 1:**
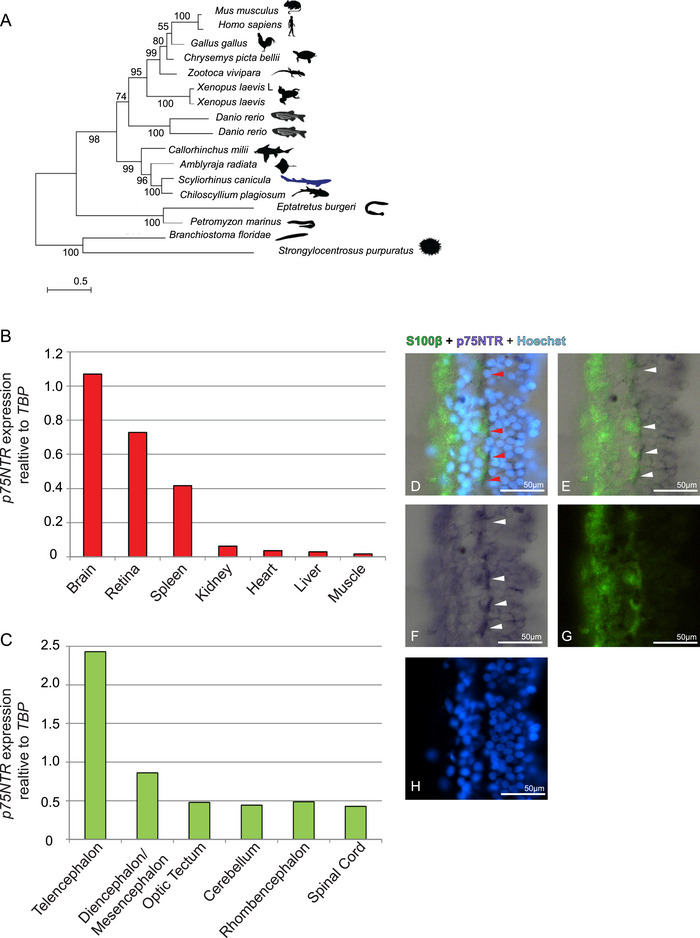
p75NTR is evolutionary conserved. Phylogenetic tree of p75NTR peptidic sequences, *Scyliorhinus canicula* is highlighted in blue (A); p75NTR expression in seven tissues of *S. canicula*, specifically the brain, retina, spleen, kidney, heart, liver, and muscle, is relative to the housekeeping TBP (B); a single specimen was assessed. p75NTR expression in the different areas of the *S. canicula* brain, relative to the housekeeping TBP, a single specimen was assessed (C). S100β IHC on p75NTR ff‐ISH of *S. canicula* retina: the p75NTR‐positive cells (D–F, red, white arrows) colocalize with the glial marker S100β (D–E; G). Nuclei are counterstained in Hoechst (D–H).

To characterize the expression of p75NTR in *S. canicula*, we first investigate its molecular evolution in Deutrostomes by reconstructing a phylogram of its protein sequences. We collected all the Chondrichthyan class peptidic p75NTR sequences from NCBI and Ensembl genome browser. We then discarded partial‐ and low‐quality sequences, ending up with a total of p75NTR complete peptidic sequences from four species, three belonging to *Elasmobranchii* subclass (*S. canicula*, *Chiloscyllium plagiosum*, *Amblyraja radiata*) and one to the *Holocephali* subclass (*Callorhinchus milii*). For the phylogenetic analysis, we used the echinoderm *Strongylocentrotus purpuratus*, the basal chordate *Branchiostoma floridae*, and two agnatha (*Petromyzon marinus* and *Eptatretus burgeri*) as outgroups and several Gnathostomata classes as ingroups: *Danio rerio* as representative teleost, which presents a duplication of p75NTR origination from teleost‐specific whole genome duplication (WGD), *Xenopus laevis* as a representative amphibian, *Zootoca vivipara* and *Chrysemys picta bellii* as representative reptiles, *Gallus gallus* as representative bird, and *Homo sapiens* and *Mus musculus* as representative mammals. We deliberately selected the best quality genomes of each class in order to minimize sequencing errors and/or gaps that could affect the analysis. The phylogram of p75NTR recapitulates the known phylogeny of these taxa with the Chondrichthyan sequences basal to all other vertebrata (Figure 1A), reinforcing the value of Chondrichthyes as the basal class of vertebrates. We confirmed p75NTR expression in the brain of *S. canicula* by qRT‐PCR and compared expression levels of p75NTR across several tissues, including the retina (Vecino et al. 1998; Carmignoto et al. 1991; Schatteman et al. 1988; Zanellato et al. 1993) and spleen (Yamamoto et al. 1996; Pérez‐Pérez et al. 2003; Ernfors et al. 1988; Laurenzi et al. 1994), which are known to express p75NTR in both adult and developing mammals. The highest expression of p75NTR was detected in the brain, followed by retina and spleen that showed higher expression as compared with the other tissues tested (kidney, heart, liver, and muscle) (Figure 1B). We further investigated regional variations in brain p75NTR expression by qRT‐PCR: The most abundant p75NTR‐expressing region of adult *S. canicula* is the telencephalic region, followed by diencephalic/mesencephalic areas, whereas optic tectum (OT), cerebellum, rhombencephalon, and spinal cord showed relatively low levels of p75NTR. This is in‐line with human p75NTR expression in the brain, as the most preponderant p75NTR‐expressing regions are the mammalian brain's two telencephalic regions: piriform cortex and basal ganglia (https://www.proteinatlas.org/ENSG00000064300‐NGFR/brain).

We investigated conservation of p75NTR expression in the retina of *S. canicula* with particular detail since both bulk‐RNA analysis and single‐cell RNAseq reported high p75NTR expression in the human retina localized to Müller glia cells (Li et al. 2023). In situ hybridizations detected p75NTR expression in a population of retinal cells scattered in the inner nuclear layer (Figure 1D–F, white and red arrows). We confirmed the identity of these cells as Müller glia by means of an S100β antibody known to label glial cells in *S. canicula* (Bagnoli et al. 2023) (Figure 1D–H, D red arrows). Taken together, our results showed for the first time the presence in *S. canicula* of p75NTR and its conserved expression pattern across tissues, brain regions, and in retina Müller glia.

### 3.2 p75NTR Expression in the Telencephalon

**FIGURE 2:**
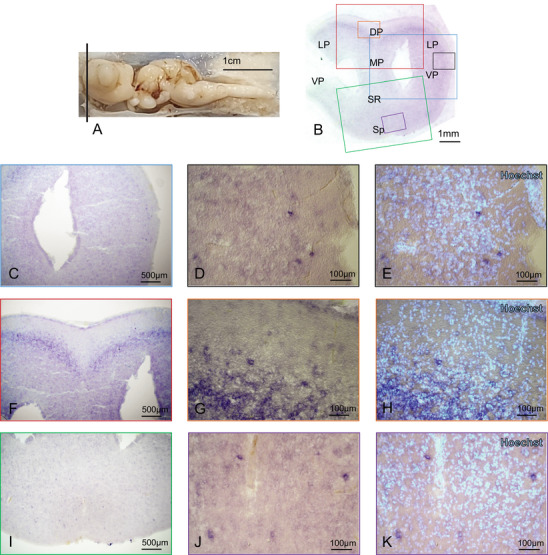
p75NTR expression in the rostral telencephalon. *Scyliorhinus canicula* brain with the black line indicating the site of the coronal sections for BDNF ff‐ISH. (A). Overview of the rostral telencephalon hybridized for p75NTR (B). p75NTR is expressed in the most rostral part of the telencephalon (B–K), with the most abundant expression concentrated in a medial streak located in DP and LP (B, F–H). Magnifications of single areas are indicated by color codes (B–K). For abbreviations, see the list. DP, dorsal; LP, lateral pallium; SR, septal region; VP, ventral pallium.

**FIGURE 3:**
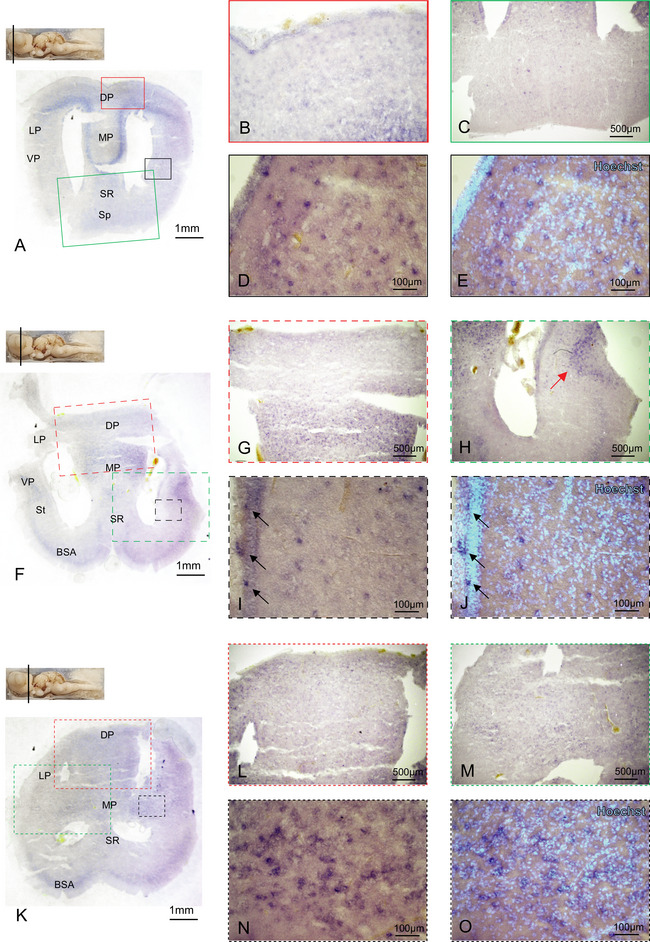
p75NTR expression in the telencephalon. Overview of the *Scyliorhinus canicula* medial telencephalon hybridized for p75NTR (A) expression in the medial and posterior telencephalon. p75NTR is expressed in the DP, LP, and VP (A–E). Magnification at the single‐cell level of p75NTR expression in the VP is shown in (D) and counterstained with Hoechst in (E). Overview of the medial telencephalon in correspondence with the olfactory bulbs hybridized for p75NTR (F). p75NTR expression is evident in the MP (F–G) and in VP (H, red arrow); magnification in (I) is counterstained with Hoechst in (J) and highlights the p75NTR‐positive cells (black arrows). Overview of the posterior telencephalon hybridized for p75NTR (K). p75NTR is expressed in the DP, LP, and MP (K–M); magnification in (N) is counterstained with Hoechst in (O). Magnifications of single areas are related to color and texture codes. For abbreviations, see the list. BSA, basal superficial area; DP, dorsal; LP, lateral pallium; SR, septal region; VP, ventral pallium.

We then proceeded to systematically localize p75NTR‐expressing cells in the brain of *S. canicula*. We performed ff‐ISH (as described in Section 2.7) on two animals (one male and one female) and detected a consistent staining pattern. Counterstaining of nuclei with Hoechst was used to identify all cells in the tissue and detect possible technical artifacts due to nonspecific staining in areas of high cellular density. Hybridized coronal sections of *S. canicula* brain show widespread expression in the most rostral part of the telencephalon (Figure 2A–K), with the most abundant expression concentrated in a medial streak located in the DP and lateral pallium (LP) (Figure 2B,F–H). This staining is consistent with high expression in the telencephalon detected by qPCR. Because of a possible retention of background due to the high cellular density, we assessed very carefully such areas with Hoechst counterstain to exclude the possibility of a background effect. Although the cellular density in such “V”‐shaped area is very high, telencephalic nuclei with the highest cellular density show a pale staining, and the specifically labeled cells stand out against the backdrop of densely packed lighter stained unspecific cells (Figure S1A–E, black arrow). As opposed to the expression of the neurotrophin BDNF (Chiavacci et al. 2023), the p75NTR receptor is abundantly expressed rostrocaudally in the DP (Figures 2B,F–H and 3A,B,F,G,K,L). The p75NTR expression is also clearly detectable in the medial pallium (MP) all along the telencephalon (Figure 2B,C, Figure S1 A–E, Figure 3F–H,K–O), whereas in the septal region (SR), its presence (Figures 2B,C,F and 3A,C,F,H) disappears in the most caudal telencephalic area (Figure S2 H–I). Other p75NTR‐expressing areas in the telencephalon are localized interspersed in the ventral pallium (VP) (Figure 3A,D–F,H), with a higher density of p75NTR‐positive cells organized in an “u”‐shaped VP area (Figure 3H, red arrow). As expected from existing data in human, rat, and mouse (Chen et al. 1996), the St also presents p75NTR‐positive cells (Figure 3F,I,J), whereas the thick band of cell in the BSA retains background staining only (Figure S2A,B,F,G). The telencephalic ventricular areas show no p75NTR expression (Figure S2 A–E), with the exception of some scattered cells in the medial telencephalic region (Figure 3,I,J, black arrows, Figure S2K,L, black arrows). p75NTR is not expressed in the posterior part of the telencephalic SR (Figure S2H,I) and is completely absent in the impaired telencephalic stalk (Figure S3). We could not detect any expression of NGF by ISH, consistent with the very low expression levels we detected previously by qPCR (Chiavacci et al. 2023).

### 3.3 p75NTR Expression in the Diencephalon and Mesencephalon

**FIGURE 4:**
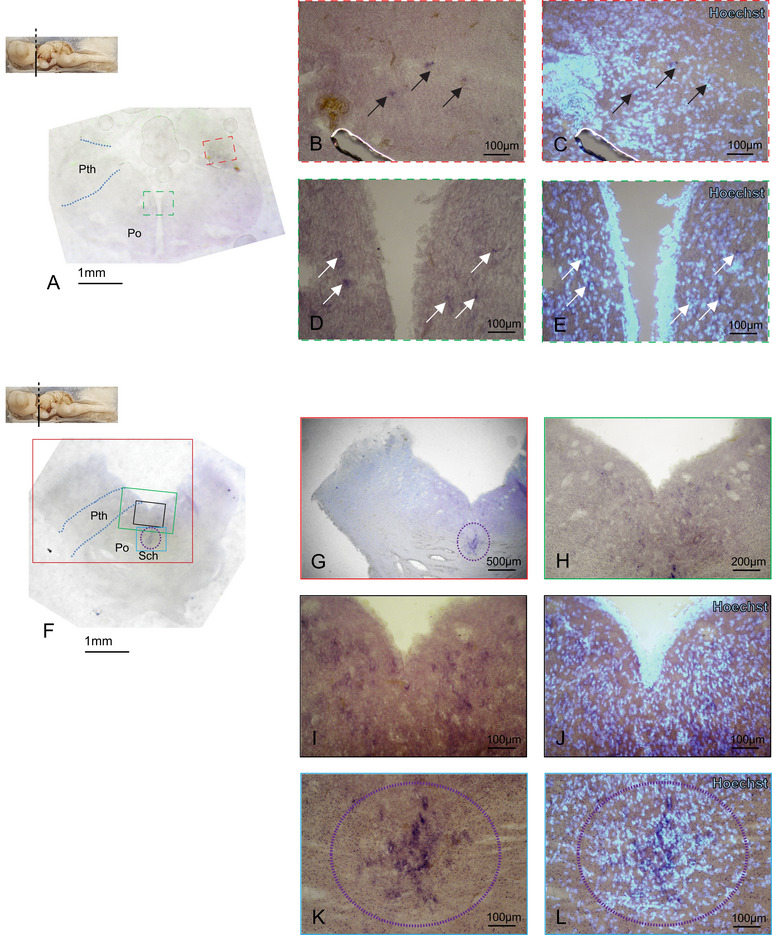
p75NTR expression in the diencephalon. Overview of the *Scyliorhinus canicula* rostral diencephalon hybridized for p75NTR (A). p75NTR is expressed in discrete cells in Pth (A–C, black arrows) and Po (A; D white arrows–E). Overview of the *Scyliorhinus canicula* diencephalon (F). p75NTR is expressed in Pth and Po (F–J) with a group of p75NTR‐expressing cells marking the Sch (F–G; K–L violet dotted circle). Magnifications of single areas are related to color and texture codes. For abbreviations, see the list. Po, preoptic area; Pth, prethalamus; Sch, suprachiasmatic nucleus.

**FIGURE 5:**
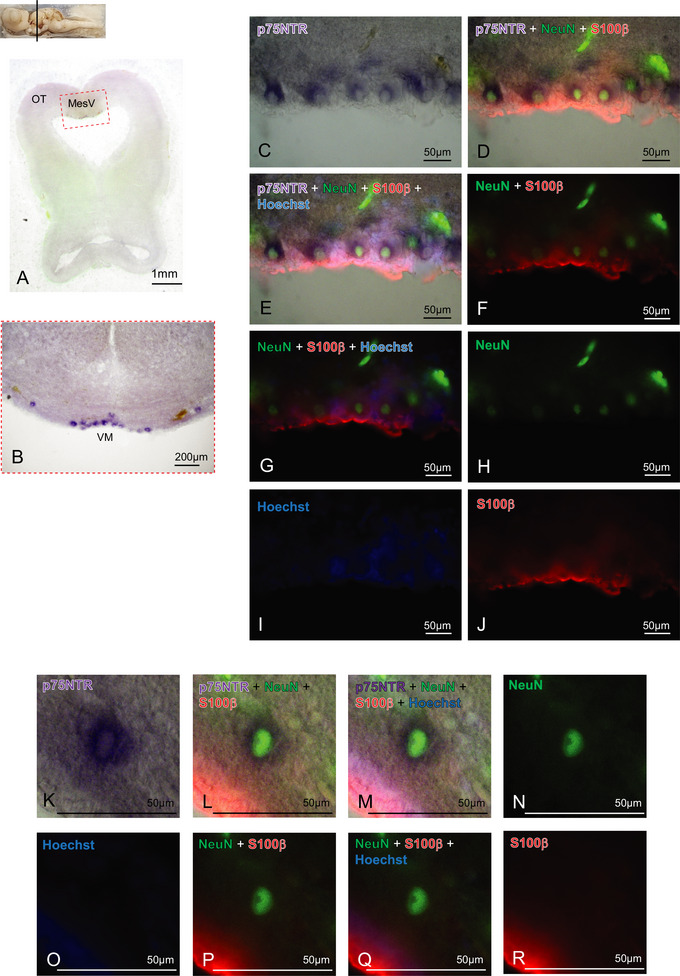
p75NTR expression in the mesencephalon. Overview of the *Scyliorhinus canicula* mesencephalon hybridized for p75NTR (A). VM giant cells are clearly detectable in MesV area and positive for p75NTR expression (A–E). Magnifications of p75NTR‐positive cells (C–E, K–M) showed their colocalization with the neuronal nuclear marker NeuN (D–H), whereas they are negative for the glial marker S100β (D–G; J; L–M; P–R). Nuclei are counterstained with Hoechst (E; G; I; M; O; Q). For abbreviations, see the list. MesV, mesencephalic trigeminal nucleus; OT, optic tectum.

In the rostral diencephalic region, the p75NTR expression is present in discrete cells distributed all along the prethalamic (Pth) (Figure 4A–C, black arrows) and preoptic (Po) (Figure 4A,D, white arrows E, F–J) areas. A prominent group of p75NTR‐expressing cells marks the suprachiasmatic nucleus (Sch) (Figure 4F,G,K,L, violet‐striped circle). In the most caudal portion of diencephalic areas, comprehensive of OT, pretectum (Pt), Th, and Pth, we did not detect any p75NTR expression cells (Figure S4A–I). The regions showing a light purple staining, for example, the PC, were proven to be areas with extremely high cellular density with background retention (Figure S4F–I). In the mesencephalic area, some very large cells in the mesencephalic trigeminal nucleus (MesV) region showed a prominent staining (Figure 5A–C). As mesencephalic motor neurons express p75NTR in chick (Von Bartheld and Bothwell 1993) and rat (Sobreviela et al. 1994), let us hypothesize their identity as trigeminal motoneurons (VM). We performed a double IHC on the p75NTR ff‐ISH, choosing the well‐established pan‐neural NeuN marker to label neuronal nuclei and with the glial marker S100‐beta, which in fish labels the entire glial cells (Bagnoli et al. 2023; D'Angelo et al. 2012). The nuclei of the giant VMs are NeuN positive and S100‐beta negative (Figure 5D–J,L–R), whereas their cytoplasm is expressing the p75NTR, as expected for the VMs (Figure 5C–E,K–M).

### 3.4 p75NTR Expression in the Rhombencephalon

**FIGURE 6:**
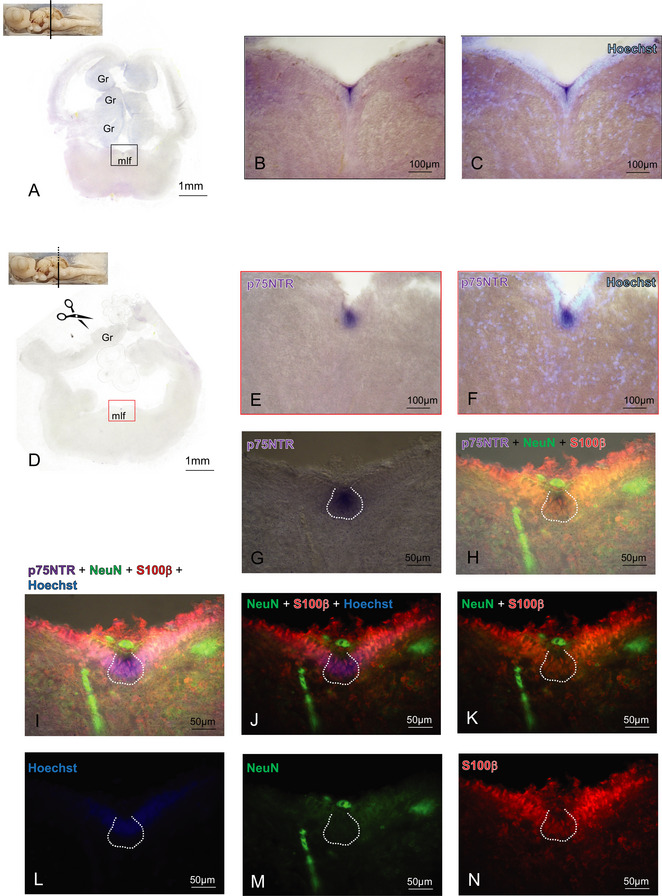
p75NTR expression in the rhombencephalon. Overview of the *Scyliorhinus canicula* rhombencephalon hybridized for p75NTR (A; D). p75NTR‐positive cells are detectable along the mlf region (A–I). Magnifications of p75NTR‐positive cells (B–C, E–I) showed their colocalization with the glial marker S100β (H–K; N), whereas they are negative for the neuronal nuclear marker NeuN (H–K; M). Nuclei are counterstained with Hoechst (I–J; L). For abbreviations, see the list. mlf, medial longitudinal fascicle.

**FIGURE 7:**
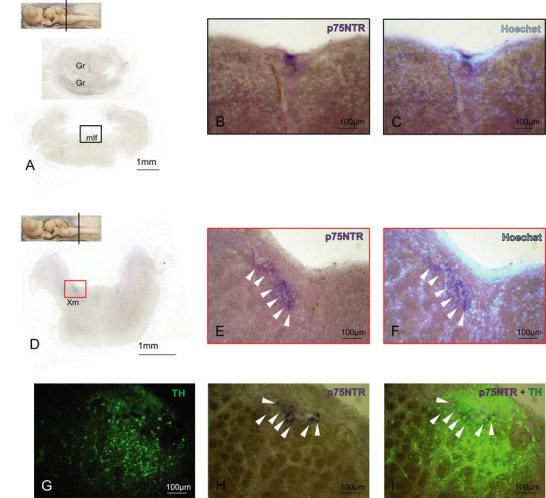
p75NTR expression in the posterior rhombencephalon. Overview of the *Scyliorhinus canicula* posterior rhombencephalon hybridized for p75NTR (A; D). p75NTR‐positive cells are detectable along the mlf region (B–C). Magnifications of p75NTR‐positive cells in the most posterior portion of rhombencephalon (D–F, H–I) showed their colocalization with the neuronal marker TH (G; I, white arrows). Magnifications of single areas are indicated by color codes. For abbreviations, see the list. mlf, medial longitudinal fascicle.

In the rhombencephalic region at the level of cerebellar granular layer (Gr), we detected a group of densely packed p75NTR‐positive cells situated in the medial longitudinal fascicle (mlf) running rostrocaudally (Figures 6A–G and 7A–C). Double IHC with NeuN and S100‐beta performed on p75NTR hybridized sections clearly showed that p75NTR cells are S100‐beta positive (Figure 6G–N) as indication of their glial nature. Due to their position in the subventricular region, we can hypothesize that such cells are of some ependymal nature. Moving caudally along the rhombencephalon, we then detected a group of p75NTR‐positive cells situated all along the DP vagal motor nucleus (Xm) (Figure 7D–F,H). IHC performed on hybridized sections showed that these cells are positive for tyrosine hydroxylase (Figure 7G–I, white arrows), thus identifying them as vagal motor neurons.

### 3.5 p75NTR Molecular Function Is Evolutionary Conserved Among Vertebrates

**FIGURE 8:**
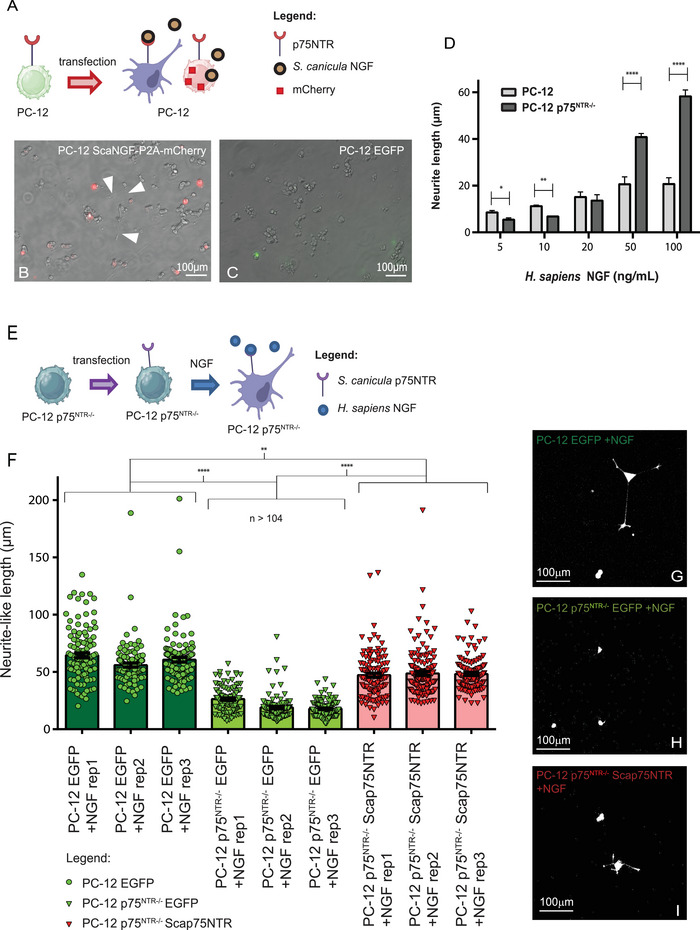
p75NTR is functionally conserved among vertebrates. Differentiation assay schematic representation: *Sca*NGF mCherry plasmid was transfected in wild‐type PC‐12 cells (A). Nine days post‐transfection some PC‐12 cells differentiated neurite‐like processes in contrast with the EGFP‐transfected controls (B, white arrows; C). Dose‐dependent response curve of wild‐type and p75^NTR−^/^−^ PC‐12 to human NGF; the wild‐type PC‐12 reached the saturation of the response to NGF signaling at 50 ng/mL of NGF, whereas the PC‐12 p75NTR^−^/^−^ showed continuous increasing. At submaximal concentration of NGF, the wild‐type PC12 cells exhibited longer neurites than the PC12 p75^−^/^−^ cells with *p value < 0.05, **p < 0.01, and ****p < 0.0001 (ANOVA‐2 followed by Bonferroni post hoc test). (D) Complementation assay schematic representation: Human NGF protein was added to p75^NTR−^/^−^ transfected with *Sca*p75NTR. (E). *Sca*p75NTR is capable to partially recover the shorter neurites of p75^NTR−^/^−^ knock‐out cells. Student *t*‐test was performed between means for each treatment, and results were considered statistically significant at *p* < 0.05. (F) Representative confocal images of counted cells for each condition (G–I). Biological replicates are indicated with rep1, rep2, and rep3. NGF, nerve growth factor.

After investigating the expression pattern of p75NTR in the *S. canicula* brain, we set to investigate its function in modulating NGF signaling using the canonical model system of PC‐12 cells, which respond to human NGF by differentiating into neurons (Greene and Tischler 1982). We cloned the full‐length *S. canicula* NGF (ScaNGF) under the strong eukaryotic CMV promoter in a vector harboring the reporter P2A‐mCherry. The resulting vector is able to express ScaNGF, and transfected cells can be visualized due to the mCherry fluorophore (Figure 8A,B). In duplicated experiments, the wells containing PC‐12 cells transfected with this vector 9 days post‐transfection also contained some differentiated cells with neurite‐like processes in contrast to control cells transfected with a CMV‐driven EGFP (Figure 8C). This indicates that the ScaNGF secreted by the transfected cells was able to activate NGF signaling in neighboring cells. We then proceeded to clone the full‐length *S. canicula* p75NTR (*Sca*p75NTR) under the strong eukaryotic CMV promoter in a vector harboring the reporter P2A‐mCherry. The resulting vector is able to express *Sca*p75NTR and conveniently labels transfected cells with the mCherry fluorophore. We then took advantage of a PC‐12 p75NTR^−^/^−^ knock‐out clone (Testa et al. 2022) to assess whether *Sca*p75NTR can complement the loss of rat p75NTR (Figure 8E). We first established a dose‐dependent response curve of wild‐type and p75NTR^−^/^−^ PC‐12 to human NGF and showed that wild‐type PC‐12 cell line reached the saturation of the response to NGF signaling at 50 ng/mL of NGF (Figure 8D), whereas the PC‐12 p75NTR^−^/^−^ showed continuously increasing size of differentiated neurite‐like processes at all the concentrations of NGF we tested (up to 100 ng/mL). All the concentrations we tested were statistically significant with the only exception of 10 ng/mL human NGF (Figure 8D). Such capability of PC‐12 p75NTR^−^/^−^ to increase the neurite‐like processes at saturating concentrations was already observed in a seminal experiment previously performed by Testa et al. (2022) and is consistent with the well‐established concept that p75NTR cooperates with TrkA to create a high‐affinity binding site for NGF (Hempstead et al. 1991; Battleman et al. 1993) and that an NGF mutant unable to bind p75NTR is three to four times less potent than wild‐type NGF in promoting neuronal survival at submaximal doses (Rydén et al. 1997). We therefore chose the 10 ng/mL sub‐optimal concentration of NGF to perform the complementation assay and measure neurite‐like processes in PC‐12 of wild‐type and p75NTR^−^/^−^ background. PC‐12 p75^NTR−^/^−^ knock‐out cells (Testa et al. 2022) were transfected with *Sca*p75NTR vector, and then human recombinant NGF protein was added to the culture media (Figure 8). Results showed that p75^NTR−^/^−^ knock‐out cells emit shorter neurites than WT PC‐12 in a statistically significant manner (*p* value < 0.0001) when human NGF is present, and this deficit is almost recovered by transfection with *Sca*p75NTR, even if the recovery is not complete (statistical significance *p* value < 0.01) (Figure 8F–I). These results highlighted the capacity of *S. canicula* p75NTR to compensate for p75NTR loss in mammalian cells, unveiling its functional conservation during the evolution of vertebrates.

## 4 Discussion

Here, we report on the first study of p75NTR in a Chondrichthyan. Chondrichthyes are of particular interest as they represent the most basal vertebrate taxon with a complete set of four neurotrophins. We identified remarkable conservation of p75NTR at the level of expression patterns and molecular function. Expression of p75NTR is restricted to a few specific neuronal populations in the mammalian brain and is prominent in the cholinergic neurons in the septum and St, the piriform cortex, the trigeminal mesencephalic neurons (Mufson et al. 1992; Jacobs and Miller 1999; Henry et al. 1993; Koh et al. 1989; Yan and Johnson JR. 1989), and the Müller glia cells of the retina (Hu et al. 1998; Ding et al. 2002). Studies on p75NTR expression in the brain in non‐mammalian vertebrates are very limited, but the expression of p75NTR in mesencephalic neurons of the trigeminal complex was reported in chick embryos (Von Bartheld and Bothwell 1993). During development, the binding of NGF to p75NTR induces apoptosis of these neurons that express TrkB, but not TrkA (Davey and Davies 1998). The role of p75NTR in adult trigeminal mesencephalic neurons is not investigated. In the retina, p75NTR expression in Müller glia is known to negatively regulate neuronal survival both during development and after injury (reviewed in Garcia et al. (2017)). For example, inhibition of p75NTR potentiates the neuroprotective effects of NGF after optic nerve injury in adult rats (Lebrun‐Julien et al. 2009) and improves survival of photoreceptors after light injury (Harada et al. 2000). We observed prominent expression of p75NTR in both mesencephalic trigeminal neurons and Müller cells, indicating a striking conservation across vertebrates and strongly suggesting that these cells were expressing p75NTR and were responding to NGF in the last common ancestor of gnathostomes. The prototypical p75NTR+ cells in the mammalian brain are the cholinergic neurons of the basal forebrain. We could not investigate the expression of p75NTR in the orthologous cells of sharks, since these cells are not described in sharks. We observed, however, expression in scattered cells in the telencephalon, and qPCR analysis identified the telencephalon as the brain region with the highest p75NTR expression. Expression was also observed in a subset of TH+ neurons in the medulla that may correspond to a subset of noradrenergic neurons of the nucleus tractus solitarius and area postrema. Expression of p75NTR was also observed in some ependymal glial cells.

The conserved expression pattern of p75NTR suggests a conserved molecular function. We address this issue directly. We first produced *Sca*NGF and tested its biological activity on PC12 cells. Amino acid sequence of NGF is moderately conserved, but simulations of *Sca*NGF structure revealed extensive structural conservation. Accordingly, *Sca*NGF induced neuronal differentiation of PC‐12 cells, indicating conservation of its molecular function. Similarly, *Sca* p75NTR was able to complement the lack of p75NTR in a mutant PC‐12 line. More specifically, p75NTR increases the affinity on TrkA for NGF. Accordingly, p75NTR^−^/^−^ PC‐12 cells retain responsiveness to NGF but require higher dosages for saturating action. Expression of *Sca*p75NTR restores dose‐responsiveness in p75NTR^−^/^−^ PC‐12 cells, indicating that *Sca*p75NTR can increase affinity for NGF of rodent TrkA. Whether *Sca*p75NTR can elicit correct signaling in the absence of TrkA remains to be investigated.

All these data indicate functional and biological conservation of NGF‐p75NTR axis across vertebrates.

## Author Contributions

**Elena Chiavacci**: conceptualization, investigation, methodology, visualization, writing–review and editing, writing–original draft. **Roberta Camera**: investigation, writing–review and editing. **Mario Costa**: resources, writing–review and editing. **Baldassare Fronte**: resources, writing–review and editing. **Eva Terzibasi Tozzini**: conceptualization, funding acquisition, project administration, resources, writing–review and editing. **Alessandro Cellerino**: conceptualization, funding acquisition, project administration, supervision, validation, writing–review and editing. All authors have read and agreed to the published version of the manuscript.

## Ethics Statement

The authors certify that animal experiments were carried out in accordance with European Communities Council Directive of November 24, 1986 (86/609/EEC) and approved by the Italian Ministry of Health (Cod. B290E.N.TU2).

## Conflicts of Interest

The authors declare no conflicts of interest.

## Supporting information



Figure S1. p75NTR expression in the rostral telencephalon.
Figure S2. p75NTR expression in the telencephalon.
Figure S3. p75NTR expression in the posterior telencephalon.
Figure S4. p75NTR expression in the mesencephalon.

## Data Availability

The data that support the findings of this study are available from the corresponding author upon reasonable request.
