# Supplementary material for: Nerve Growth Factor Receptor (NGFR/p75NTR) of the Small‐Spotted Catshark (Scyliorhinus canicula): Evolutionary Conservation and Brain Function
Source: J Comp Neurol. 2025 Apr 12;533(4):e70049. doi: 10.1002/cne.70049 (PMC11993139; doi:10.1002/cne.70049)
Supplement: Supplementary file 1 — Figure S1. p75NTR expression in the rostral telencephalon. Figure S2. p75NTR expression in the telencephalon. Figure S3. p75NTR expression in the posterior telencephalon. Figure S4. p75NTR expression in the mesencephalon. [file CNE-533-e70049-s001.pdf]

S 1

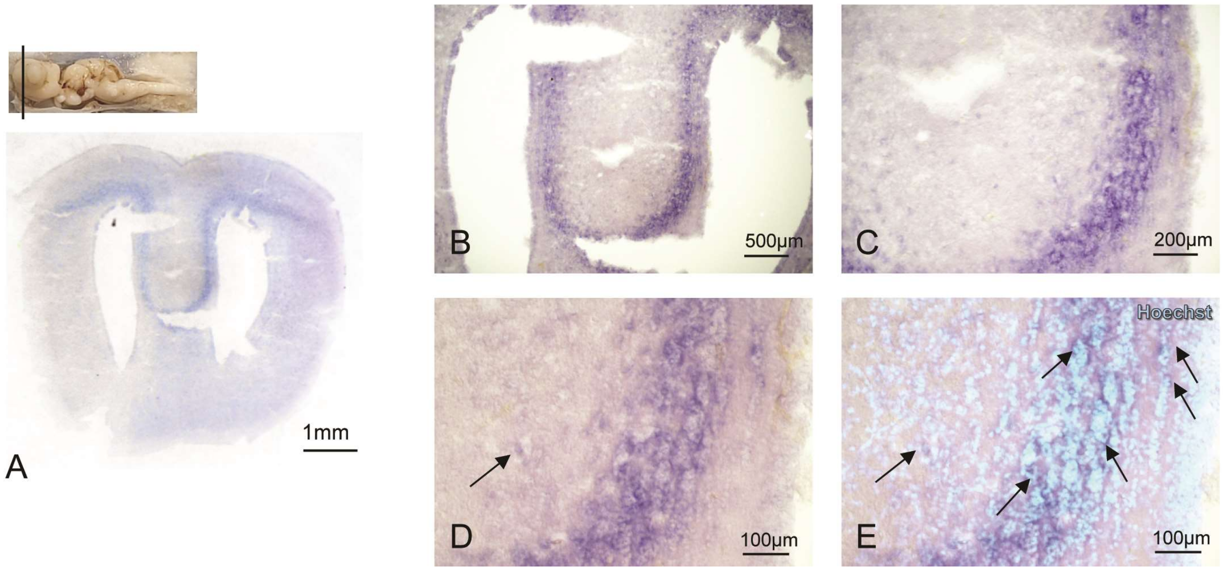

Figure S1: p75NTR expression in the rostral telencephalon. Overview of the rostral telencephalon hybridized for p75NTR (A). Thanks to the Hoechst counterstaining the V-shaped background due to cell density is evident compared to the hybridized single-cell (B-E).

S 2

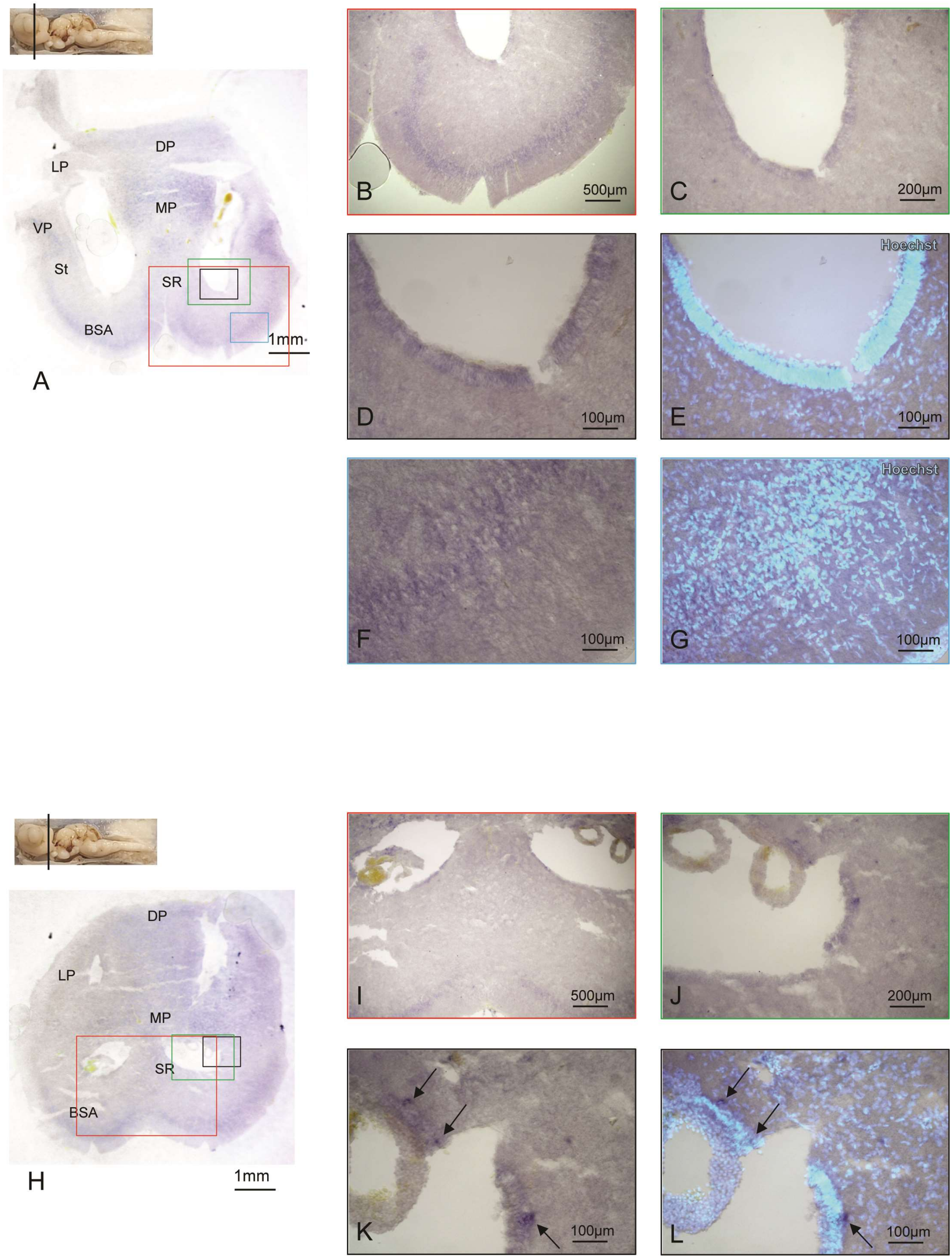

Figure S2: p75NTR expression in the telencephalon. Overview of telencephalon (A) and posterior telencephalon (H) hybridized for p75NTR. Thanks to the Hoechst counterstaining the background due to cell density (E-G) is evident in the BSA and ventricles compared to the

hybridized single-cell (K-L black arrows). Magnifications of single areas are indicated by color codes. For abbreviations, see the list.

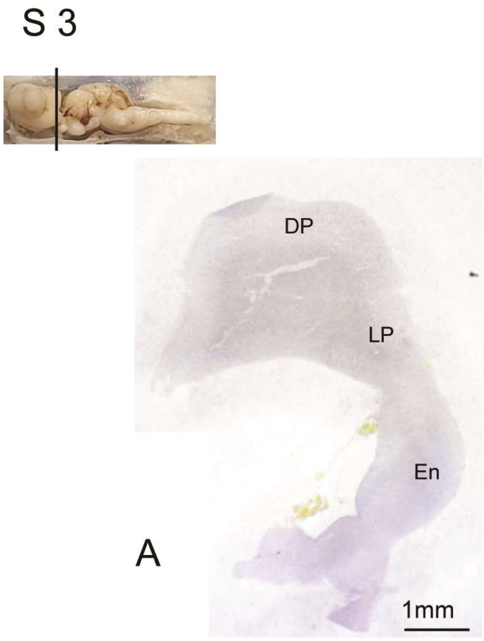

Figure S3: p75NTR expression in the posterior telencephalon Overview of the *S. canicula* posterior telencephalon hybridized for p75NTR (A). p75NTR expression was not detected. For abbreviations, see the list.

S 4

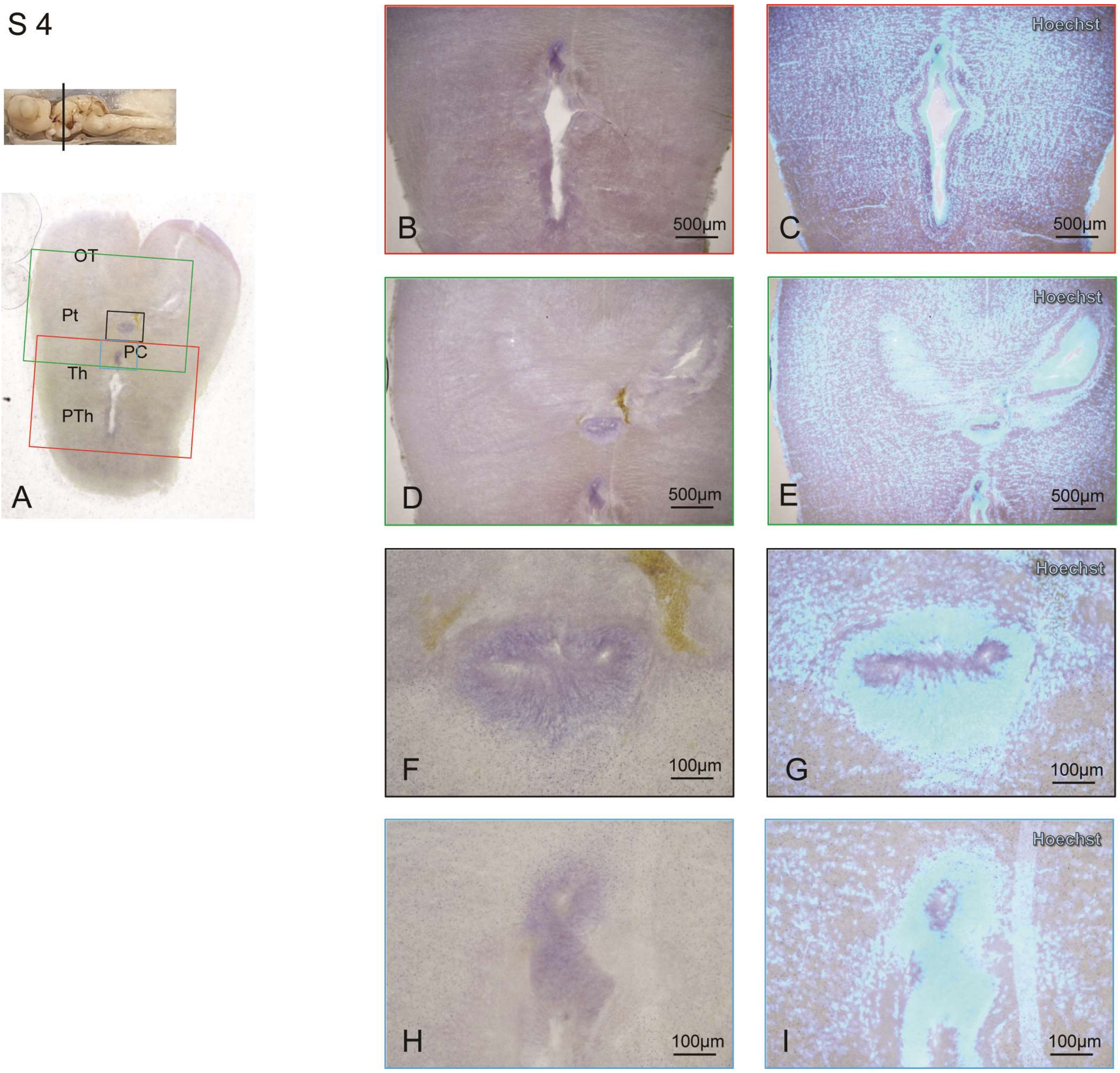

Figure S4: p75NTR expression in the mesencephalon. Overview of the *S. canicula* mesencephalon hybridized for p75NTR (A). p75NTR expression was not detected while thanks to the Hoechst counterstaining the background due to cell density was evident (B-I). Magnifications of single areas are related to color codes. For abbreviations, see the list.
